# Therapeutic Approaches Interfering with Nuclear Localization Signals: An Emerging Strategy for CNS-Related Diseases

**DOI:** 10.2174/011570159X384321250627071259

**Published:** 2025-07-14

**Authors:** Margrate Anyanwu, Matteo Giannangeli, Alessandra Gianoncelli, Giovanni Ribaudo

**Affiliations:** 1 Department of Molecular and Translational Medicine, University of Brescia, Viale Europa 11, Brescia, 25123, Italy

**Keywords:** CNS, karyopherins, cancer, neurodegeneration, small molecules, peptides, antivirals

## Abstract

Although medicinal chemistry is constantly looking for new therapeutic approaches against pathological conditions affecting the central nervous system (CNS), such as neurodegeneration and cancer, this quest has not been fully successful yet. The lack of understanding of all the complex mechanisms underlying these conditions makes the identification of new effective drugs challenging. A wide variety of pathophysiological events are regulated at both nuclear and cytoplasmic levels, and in this context, targeting the shuttle system composed of the karyopherin superfamily and their cargoes may provide an alternative strategy. Molecular recognition is highly specific and strictly related to the presence of special “tag” regions, known as nuclear localization signals, that are localized in the amino acid sequences of cargoes. Importantly, their trafficking is involved in various pathophysiological processes, including CNS diseases. Curiously, although this system has been studied intensively, much remains to be discovered to date. Throughout the years, drug discovery allowed the identification of small molecules and peptides able to target karyopherin-cargo complexes to provide new potential pharmacological treatments. Indeed, the first examples of drug candidates targeting this mechanism that reached clinical trials are appearing in the literature. With this mini-review, this study aims at presenting an updated overview on the most recent reports investigating the use of the karyopherin shuttle system as a new therapeutic target especially for CNS-related diseases.

## 1 INTRODUCTION

### 1.1 CNS Drug Discovery: An Ongoing Challenge

This article has highlighted the importance of the karyopherin-dependent system, with the aim of identifying new possible targets for the development of new therapeutic approaches in the field of central nervous system (CNS) diseases. For this reason, a literature search was performed and 150 publications were retrieved thanks to query strings containing the words “karyopherin”, “phosphodiesterase”, “CNS”, “drug discovery”, “protein-protein interaction” and their combinations. Neurological disorders currently attract special attention from the perspective of drug treatment. Although methods for drug research and development are constantly evolving, identifying molecules that can target the CNS remains an open challenge [1-3]. For every disease, it is difficult to discover effective and safe drugs, and this depends on a combination of variables. In the case of CNS diseases, this is amplified by the fact that a full comprehension of the complex underlying biology is missing. Further, the absence of specific biomarkers is reflected by a high failure rate that drug identification encounters in this area compared to its peripheral counterpart [4]. Moreover, already available approaches in most cases are limited to giving relief to patients through symptomatic treatments rather than completely resolving the pathological conditions [3, 4]. The World Health Organization (WHO) has stated that brain disorders fall within the top 10 causes of mortality worldwide and that, within these conditions, neurodegenerative disease (NDDs) represent the main group [5].

Both cancer (*e.g*., glioma) and NDDs (*e.g*., Parkinson’s and Alzheimer’s diseases, PD and AD, respectively) exert a destructive force at the level of the anatomical components, and a variety of symptoms reflect this. Although these differ in incidence, mortality, and etiology, few are the markers that allow their identification. In this case, one of the main reasons stems from the localization of affected cell populations that make the availability of diagnostic markers, to date, limited [6-8]. The complexity in efficiently treating CNS cancer and NDDs is supported by their resistance to therapy, making them more challenging to treat. Because of this, few drugs have reached the market to date.

Successful drug discovery and development depend on a detailed knowledge of pathophysiological mechanisms. In this context, one of the most difficult hurdles to overcome is the location of CNS targets that are protected by the blood-brain barrier (BBB) [9]. BBB is a vascularized semi-permeable barrier acting as a protection system for CNS functional units like neurons, astrocytes, and pericytes, which are constantly supplied by nutrients, deprived of waste, and protected by exogenous molecules. The position of BBB allows it to have a pivotal role in the CNS, thus being one of the most important aspects to consider in drug design from medicinal chemistry studies. Indeed, the ability of ligands to cross the BBB is a crucial parameter to consider in the drug discovery process [10-14]. The fine regulation that the BBB does is given by active and passive transport. Although passive diffusion of small lipophilic molecules is a major mechanism of penetration for CNS drugs, often efflux pumps send back the compounds, preventing their access to the CNS [15]. Usually, these mechanisms rely on several transporters belonging to the ATP-binding cassette (ABC) transporter family, such as P-glycoprotein (P-gp), breast cancer resistance protein (BCRP), and members of the multidrug resistance protein (MRP) subfamily, which are highly expressed and determine an active transport that leads to a reduction in the effective concentration of molecules within the CNS [16-18]. From a medicinal chemistry point of view, capturing every feature that can enable the effective migration of molecules into the brain is crucial to the progress of CNS drug discovery [19, 20]. For CNS ligand design, it is relevant to consider several aspects that involve physicochemical properties (*e.g*., solubility and size), pharmacokinetics, and pharmacodynamics, and try to assess whether they are substrates for efflux pumps or not. Over the years, several rules have been proposed to standardize and summarize the features that ligands should fulfill to access into CNS by crossing the BBB [21]. This exploration led to the definition of a subgroup defined as “CNS chemical space,” which has seen several changes over the years. The first benchmark against which the chemical space for CNS drug candidates was explored was Lipinski's rule of five, which summarizes the features that a molecule must possess to be associated with oral bioavailability [22, 23]. Although this rule allowed for to identify of a number of promising compounds with good approximation, analysis of the CNS chemical space has led to the optimization of the parameters needed for CNS drugs, resulting in the application of stricter rules. Indeed, these drugs occupy a considerably more limited chemical space compared to drugs directed at the peripheral counterparts [20, 24]. One of the most important physicochemical properties that this allowed us to find was the lipophilicity. An increase of this property is reported to be an increment of the *in vitro* potency. In contrast, extremely high lipophilicity values fall back to low solubility and an increase in the number of unwanted activities [25]. Then, the hydrogen bonding potential for a drug is essential since an increase in it reduces lower passive permeability, and this runs in parallel with a higher possibility of recognition by P-gp efflux pumps [26]. Further, even the number of hydrogen bond acceptors and donors (respectively, HBA and HBD) is pivotal for CNS targeting. In this context, Pajouhesh and Lenz proposed the following guidelines for successful CNS drug candidates: HBD < 3, HBA < 7, and total hydrogen bonds < 8 [27]. Accordingly, a reduction in HBD capacity is one of the most frequently reported medicinal chemistry strategies for improving brain exposure [28]. Another evaluated aspect is the polar surface area (PSA) of a small molecule that can be calculated in different ways. Molecules with a PSA > 140 Å^2^ generally have poor gut permeability, leading to low oral bioavailability [29]. However, reflecting the differences in the gut and BBB permeability as well as plasma and brain tissue binding, PSA values for CNS penetrant compounds tend to be considerably lower. A comparative analysis of marketed CNS and non-CNS drug properties led Van de Waterbeemd to propose a PSA value lower than 90 Å^2^ as a cutoff for optimal CNS exposure [30]. Based on a similar analysis performed on a different data set, Kelder suggested an even stricter cutoff of PSA comprised between 60-70 Å^2^ [31]. These parameters, discovered over the years, confirmed how the exploration of CNS chemical space still needs to find an *optimum* for treating CNS diseases [32-34]. The goal of finding new strategies that can circumvent the issue of BBB penetration to reach the CNS targets has led to the use of alternative small molecules, such as peptides, that nowadays allow to achieve pioneering results for treating CNS cancers and NDDs [35-39]. Different classes of CNS-selective peptides have been designed and their main features regards BBB crossing. Among these, there are cell-penetrating, neuroprotective, and anti-inflammatory peptides, and their main goal is to mimic specific recognition regions present in their targets. In the group of strategies employed for an alternative CNS targeting, natural and synthetic peptides are one of the most studied approaches [37, 40]. Natural peptides can come from humans, plants, and animals. Within this category, several examples can be considered, and the main ones are carnosine, amylin, RI-OR2 [41, 42], QBP1, ED11 (WO2014057484A1) [43], and their derivatives [36, 44-46]. Many identified synthetic peptides need to undergo further modifications to improve their pharmacokinetic properties before clinical application. Hence, after the target is known, it is essential to rely on structure-activity relationship (SAR) studies to characterize active binding domains of the target.

#### 1.1.1 Karyopherins as Novel CNS Targets

Several groups attempted to unravel these mechanisms, and, in this context, many pathophysiological processes were identified to deregulate intracellular transfer, thus altering cell homeostasis at nuclear or cytoplasmic levels. These two cellular compartments are known to be constantly communicating through the karyopherin (Kap) shuttle system; hence, medicinal chemists try to identify and develop efficient Kap-shuttle interfering ligands [47]. This system allows to transport macromolecules (defined as “cargoes”) with the aim of shuttling them from the cytoplasm to the nucleus or *vice versa,* depending on cell demands. Nuclear pore complexes (NPCs) components are referred to as nucleoporins, and to form a functional NPC, ~600 copies of ~30 nucleoporins are required [48]. NPCs were thought to be involved only in transport activities, but multiple studies highlighted their involvement in other biological events such as transcriptional control, small ubiquitin-related modifier homeostasis, cell cycle progression, chromatin organization, and RNA biogenesis [49].

**Fig. (1):**
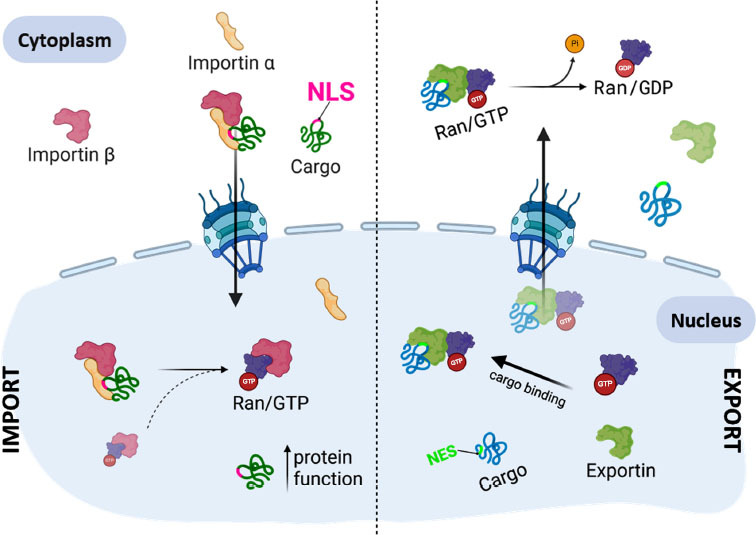
Representation of importin/exportin-mediated nucleocytoplasmic transport of macromolecules. Nuclear import of cargoes (left panel) occurs through Importin-α, which binds to the NLS within cargo proteins, then a ternary complex forms in the cytoplasm by binding to Importin β and allows the nuclear entry. Next, the binding of RanGTP and Importin β in the nucleus allows for the dissociation of the complex and protein functioning. For nuclear export (right panel), RanGTP stimulates binding of exportin to an NES-containing cargo protein in the nucleus, then the complex is exported to the cytoplasm, where hydrolysis of RanGTP to RanGDP results in complex disassembly. The figure was prepared using items from Biorender.com; licensed to Department of Molecular and Translational Medicine, University of Brescia.

The active process transports nearly all macromolecules in and out of the nucleus with the help of transport signals and the Kap protein superfamily. Kaps specifically bind transport signals found on cargoes and translocate them through the NPC channel. The most studied transport signals are found on nuclear protein cargoes. Such signals consist of short amino acid sequences called “nuclear localization sequences” (NLSs; for import) or “nuclear export sequences” (NESs; for export) that are found within their structures [50]. The Kaps family is divided into two major groups: Kaps-α and Kaps-β. The former directly recognizes NLSs and NESs on the cargoes, acting as an adaptor protein between cargoes and Kaps-β. The latter recognizes Kaps-α/cargo complexes and subsequently binds NPCs, leading to translocation events [48]. Kaps-α are classified in three subfamilies, α1 (IMPα1, IMPα8), α2 (IMPα3, IMPα4), and α3 (IMPα5, IMPα6, IMPα7), depending on differences in their amino acid sequence, defining cargo selectivity. These subfamilies generally present a conserved region containing ten helical armadillo repeats, a C-terminal domain which is involved in the exit from the nuclear environment toward the cytoplasmic one, and an N-terminal domain involved in the recognition of NLSs of the specific cargoes through the presence of the importin β binding (IBB) domain responsible for the interaction with Kaps-β [51, 52]. On the other hand, from a structural point of view, Kap-β is divided in two main regions: a common N-terminal binding site involved in the release of cargoes and a C-terminal domain characterized by 18-20 tandem huntingtin, elongation factor 3, protein phosphatase 2A, and TOR1 (HEAT) motifs composed by pairs of antiparallel α-helices connected by a turn, all organized into a flexible right-handed solenoid structure [52]. This architecture is pivotal since it gives Kaps-β the ability to bind multiple interactors, such as Kaps-α, cargoes, and intrinsically disordered proteins (IDPs) at the same time [52-55]. The directions in which Kaps-β transport their cargoes lead to their classification in three categories: importins (towards the nucleus), exportins (towards the cytoplasm) and biportins (both directions). Currently, at least twenty human Kaps-β are known, of which 10 are importins (IMP or IPO; IMPβ, Transportin/Karyopherin-β2 (KAPβ2), Transportin 2/Karyopherin-β2b, Transportin 3/Transportin-SR, IPO4, IPO5, IPO7, IPO8, IPO9, IPO11) (Table **1**), 5 are exportins (XPO; Chromatin Region Maintenance 1 CRM1/XPO1, Cellular Apoptosis Susceptibility/XPO2, XPOT, XPO5, XPO6) (Table **2**), 3 are biportins (IPO13, XPO4, XPO7) (Table **3**), while the role of the other two (RanBP6, RanBP17) has yet to be determined (Table **4**) [56]. Like most intracellular transport, Kap-mediated regulation requires energy, and in these processes, the RanGTPase family is crucial [50]. These are GTPases that function as a molecular switch, converting the active GTP-bound and inactive GDP-bound complexes (Fig. **1**) [57]. Their dual role in both cellular compartments leads to a gradient within the cell that allows the dissociation of cargoes from their shuttles both in the nucleus and cytoplasm [58, 59]. As a result, Kaps are involved in physiological processes like mitosis and gene transcription, while several studies have reported their contribution to pathophysiological events like cancer, NDDs, and viral infections [50, 52, 60, 61]. Given the importance that nuclear transport has, the involved proteins might be considered attractive druggable targets.

## 2 SMALL MOLECULES AND PEPTIDES FOR TARGETING KAPS-MEDIATED ACTIVITIES

Given these premises, regulating Kaps-mediated intracellular transport might be an alternative path to explore in the quest for new therapeutic options. As will be discussed in the following sections, some of these approaches aimed at interfering with the Kaps/cargoes recognition at different levels with both small molecules and/or peptides [62]. In particular, some of the cases that have stood out mainly relied on IMPα/β-dependent transport, on the inhibition of cargoes recognized by the IMPα/β complex, and on the inhibition of XPO-dependent transport [63, 64].

### 2.1 IMPα/β-dependent Transport Inhibitors

IMPα and IMPβ inhibitors prevent the import of cargoes. Among IMPα inhibitors, small molecules like ivermectin [65] and gossypol were reported in the literature [66, 67]. Selective targeting of IMPα5 by ivermectin was described to reduce the import of hypoxia-inducible factor 1 subunit α (HIF1α) and its related translational and angiogenic events [68]. Further, synthetic inhibitor peptides like bimax1, bimax2 [69], and N50 [70] are found to bind IMPα with high affinity (K_d_ in pM range) and mimic the natural NLS sequence of proteins [68]. The sequence of bimax1 (GSRRRRPRKRPLEWDEDEEPPRKRKRLW) and bimax2 (GSRRRRRRKRKREWDDDDDPPKKRRRLD) attempted to resemble a typical bipartite cNLS consensus region in IMPα independently of the presence of IMPβ. Results demonstrated that these peptides were able to disrupt the protein-protein interaction, interfering with Retinoblastoma-like protein 1 (p107) involved in cell cycle regulation and tumor suppression and with Protein Inhibitor of Activated STAT Y (PIASy) involved in cell proliferation and transcriptional activity [71, 72]. N50 (VQRKRQKLMP) is a synthetic peptide selective for IMPα, and in particular for IMPα5 (K_d_ in the nM range), which can mimic the NLS pattern PKKKRKV and it is reported to modulate the import of pro-inflammatory cargoes like nuclear factor kappa-light-chain-enhancer of activated B cells (NF-κB), Signal transducer and activator of transcription 1 (STAT1) and Activator protein 1 (AP-1) [70].

On the other hand, a higher number of small molecules and fewer peptides that exert their actions as IMPβ inhibitors are reported in the literature [73]. In this group, karyostatin 1A reduces the Nuclear factor of activated T-cells (NFAT) nuclear import based on IMPα/β but does not affect NFAT XPO1-dependent nuclear export in HeLa cells [74]. Furthermore, importazole (IPZ) is reported to have selectivity in myeloma cell lines since the inhibition of NF-κB nuclear import induced apoptosis. This mechanism of action (MoA) is reported to occur with a similar selectivity toward NFAT target [75]. Moreover, IPZ was known to be effective at peripheral levels, and consequently, Pathak and colleagues reported its activity in the CNS at the level of hippocampal cells derived from C57/Black 6, where IMPβ-mediated nuclear import was prevented [76, 77]. Further, IPZ reduced cell viability in glioblastoma (GBM) cell lines. In this work, both Ran-positive (VTC-103) and Ran-negative (VTC-037) primary GBM cell lines were evaluated. As expected, since RanGTPase is implicated in IMPβ-mediated transport, VTC-103 cell lines were more sensitive to IPZ [78]. Moreover, similar results were obtained on different human GBM cell lines (U-87 MG, U-251 MG, A-172 MG, and SHG-44) by inducing apoptotic events [75, 79].

**Fig. (2):**
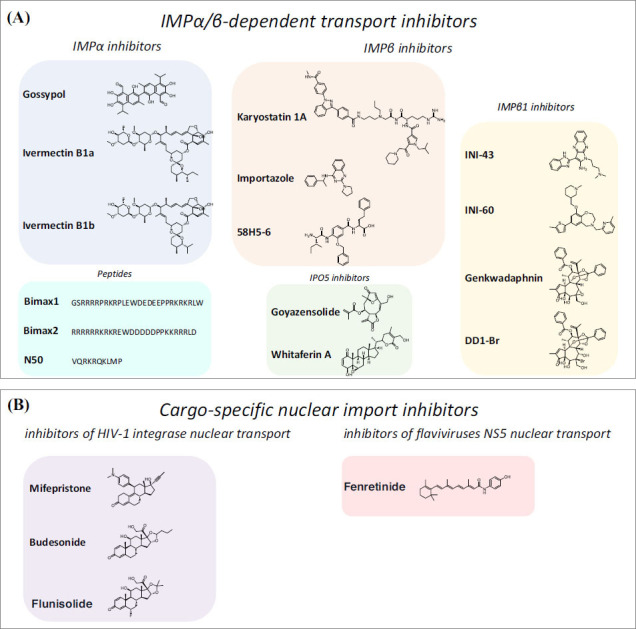
IMPα/β-dependent and cargo-specific nuclear import inhibitors. (**A**) IMPα inhibitors can be small molecules (gossypol, ivermectin B1a and B1b) or peptides (bimax1, bimax2, N50). IMPβ can be divided into non-selective, IMPβ-specific or selective for IPO5. (**B**) Cargo-specific nuclear import inhibitors can be classified into inhibitors of HIV-1 integrase nuclear transport (mifepristone, budesonide, flunisolide) and inhibitors of flavivirus NS5 nuclear transport (fenretinide).

Other small molecules like INI-43 [80], INI-60 [81], 58H5-6 [82], genkwadaphnin (DD1), and its derivative DD1-Br were reported [83, 84]. The latter, a brominated derivative of the natural diterpene DD1, has been recently reported to be able to significantly reduce the nuclear import of transcription factors such as Cellular myelocytomatosis oncogene (c-MYC), E2 transcription Factor 1 (E2F1), androgen receptor (AR), Forkhead box protein M1 (FOXM1), and coactivators like proliferating cell nuclear antigen (PCNA), cAMP response element-binding protein (CREBBP) and PARK7 Parkinsonism associated deglycase (PARK7) by targeting IMPβ1 in C4-2B prostate cancer cell line [84]. A novelty in the field is represented by goyazensolide, which is a natural sesquiterpene lactone that was identified as the first covalent IPO5 inhibitor [85]. Interestingly, this compound was found to be effective in reducing cell viability in human GBM cell lines U-87 MG and T98-G and was able to successfully cross a BBB mimic membrane, showing potential as a candidate for GBM treatment [86]. Recently, a steroidal lactone, withaferin A, has been reported as another covalent binder of IPO5, on which more studies need to be done to unravel its MoA (Fig. **2A**) [86].

#### 2.1.1 Cargo-specific IMPα/β Inhibitors: an Application for Viral Therapeutics

One of the explored fields of application for cargo/IMPα/β complex disruption regards viral proteins. Several drugs were identified to prevent the entrance of viral proteins into the host nuclear environment, thus inhibiting viral replication. In this regard, several synthetic steroids like mifepristone, budesonide, and flunisolide belong to this group. Their MoA relies on targeting the NLS region of human immunodeficiency virus type 1 (HIV-1) integrase, blocking its nuclear import, and potentially inhibiting the transport of the HIV pre-integration complex into the nucleus [87]. Further, fenretinide is a promising antiviral effective on flaviviruses, like Dengue, Zika virus, and West Nile viruses, by targeting their non-structural protein 5 (NS5) and blocking their nuclear translocation (Fig. **2B**) [88-90].

### 2.2 XPO-dependent Transport Inhibitors

The XPO-dependent transport inhibitor class includes drugs that interfere with the nuclear export process. Most of these compounds specifically target XPO1, which is currently the most characterized exportin. From the structural point of view, the XPO1 binding site was characterized in 2010 by Güttler and colleagues, and it was found that a set of five pockets (φ0-φ4) compose the binding site and recognize different NES peptides [90]. However, a new pocket was discovered recently. It is called φ1* since it is found below φ1 [91].

The first compound reported as an XPO1 inhibitor is leptomycin B (LMB), a natural polyketide that selectively alkylates Cys528 of human XPO1, thus inhibiting its nuclear export activity [92, 93]. Furthermore, LMB is also reported to inhibit NF-κB activity by blocking an NF-κB inhibitor (IκB-α) degradation *via* XPO1 inhibition in HeLa cells [94, 95]. Due to high toxicity issues, including nausea, vomiting, severe anorexia, and malaise, LMB was discontinued from the Phase I clinical trial that enrolled patients with a wide variety of cancer types [96-98]. Anguinomycins C and D are natural polyketides structurally like LMB that inhibit XPO1 through the same mechanism as LMB [99]. Goniothalamin and its derivatives covalently bind XPO1 in a hydrophobic cleft that recognizes the NESs of its cargoes [100, 101]. Moreover, it is reported that these compounds in HeLa cells inhibit the cytoplasmic Right open reading frame 2 protein kinase (Rio2-kinase) involved in ribosome biogenesis, which relies on nuclear export [99, 100, 102]. Interestingly, LMB, anguinomycins, goniothalamin, and their derivatives are characterized by the presence of an unsaturated δ-lactone ring that acts as a Michael acceptor, which is involved in a Michael addition reaction with the thiol moiety of Cys528, a nucleophilic group, thus acting as a Michael donor. This was also predicted through computational studies (*e.g*., covalent docking), providing further evidence of the importance of the lactone ring in XPO1 covalent inhibitors [99]. Differently, other small molecules like curcumin and its analogue dibenzylideneacetone are other compounds identified as XPO1 inhibitors that behave as Michael acceptors, allowing for covalent interaction with Cys528. Related biological events include the block of NF-κB, myeloid differentiation protein 2 (MD2), protein kinase B/mammalian target of rapamycin (Akt/mTOR), and STAT3 signaling pathways, inhibition of Forkhead box protein O1 (FOXO1) export in the cytoplasm, and upregulation of p73 and p27 [103-105]. Interestingly, a hydrogenated metabolite of curcumin, octahydrocurcumin (OHC), showed anticancer activity in an H22 tumor-bearing mouse model by inducing cell apoptosis through p53 upregulation and murine double minute 2 (MDM2) downregulation. However, OHC distinguishes itself by not being a Michael acceptor and not a covalent binder with Cys528, thus requiring more studies to unravel its MoA [106]. Curcumin was also tested in combination with proteasome inhibitors (bortezomib, carfilzomib, or ixazomib) or immunomodulatory drugs against multiple myeloma (thalidomide, lenalidomide, or pomalidomide) in 15 dexamethasone intolerant multiple myeloma patients, showing a better tolerance and diarrhea as only side effect in two patients, that was managed by lowering the dose or temporally suspend the therapy [98, 107]. Plumbagin, a natural naphthoquinone, is another XPO1 covalent inhibitor selective for Cys528 binding. It prevents NF-κB activation and IκBα degradation through XPO1 inhibition, and can disrupt XPO1 complexes with FOXO1, p21, p53 and p73 [108]. Plumbagin is also being investigated as a potential therapeutic agent in prostate and non-small cell lung cancer [109, 110]. Furthermore, CBS9106 (felezonexor) and its derivative S109 are the first reversible XPO1 covalent inhibitors selective for Cys528 reported in the literature [97, 111]. Due to their relevance, several clinical studies were done and, in this context, felezonexor was completed in Phase I clinical trials (NCT02667873, NCT05795192, NCT05522868) where patients affected by solid tumors were involved [112-114]. The patients involved in these clinical trials showed adverse events, including nausea, vomiting, fatigue, decreased appetite, diarrhea, acute renal injury, and neutropenia [98, 115].

Avoiding XPO1 permanent inhibition is preferable since the majority of highly toxic effects could be addressed to covalent binding [96, 97]. Subsequently, a new series of compounds called selective inhibitors of nuclear export (SINEs), which are reversible covalent inhibitors, were developed. KPT-127, KPT-185, KPT-205, KPT-225, KPT-227, KPT-249, KPT-251, KPT-276, KPT-301, KPT-330 (selinexor), KPT-335 (verdinexor), and KPT-8602 (eltanexor) belong to this class and are characterized by the presence of an N-azolylacrylate scaffold, that was previously found in PKF050-638, a small molecule that inhibits the XPO1-dependant nuclear export of HIV-1 Rev, a protein which is required for HIV replication [116, 117]. SINEs have been the most successful drug candidate class in this context so far. Indeed, selinexor has received the Food and Drug Administration (FDA) approval for the treatment of refractory multiple myeloma and diffuse large B-cell lymphoma [118-121]. Moreover, SINEs, especially selinexor and eltanexor, are being investigated *in vitro* and/or *in vivo* for their application against solid cancers. In this context, KPT-127 and KPT-205 were investigated for pancreatic and prostate cancers, KPT-225 and KPT-301 for prostate cancers; KPT-227 for pancreatic cancers; verdinexor for neuroblastoma and esophageal cancers and KPT-185, KPT-251, KPT-276, selinexor, and eltanexor for various cancers [122]. Furthermore, SINEs are investigated in brain cancers, and eltanexor has been highlighted as a promising anti-PD compound [123, 124]. In particular, Liu *et al*. [124] found that eltanexor inhibits the activation of the NF-κB pathway and the NLRP3 inflammasome by inhibiting the nuclear transport of p65 NF-κB subunit, an IMPα3 and IMPα4 cargo, thus resulting in an attenuation of Lipopolysaccharides-induced peripheral inflammation and 1-methyl-4-phenyl-1,2,3,6-tetrahydro pyridine (MPTP)-induced neuroinflammation. Moreover, selinexor has been involved in many clinical trials against glioblastoma, the most common brain cancer, in combination with standard of care therapy with encouraging results (NCT04216329, NCT04421378, NCT01986348, NCT05432804) [125-130]. However, the administration of selinexor, a first-generation SINE, is associated with many side effects, such as thrombocytopenia, fatigue, nausea, anemia, hyponatremia; while eltanexor, a second-generation SINE, show a similar adverse effect profile, but with lower incidence and severity of fatigue, nausea and hyponatremia, thus the development of non-covalent inhibitors has become important to try to reduce these effects [131, 132].

**Fig. (3):**
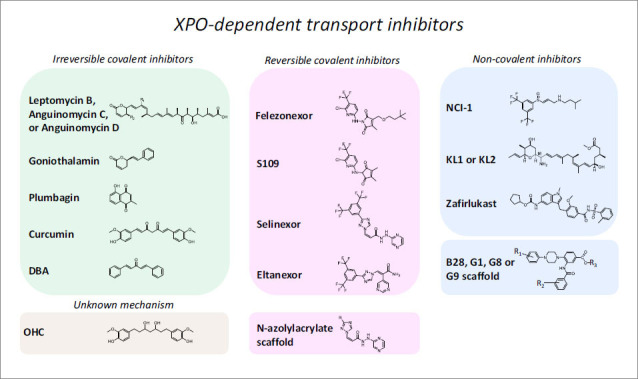
XPO-dependent transport inhibitors. They can be divided into irreversible (leptomycin B (R_1_=C_2_H_5_, R_2_=CH_3_), anguinomycin C (R_1_=CH_3_, R_2_=H) anguinomycin D (R_1_=C_2_H_5_, R_2_=H), goniothalamin, plumbagin, curcumin, DBA) and reversible (felezonexor, S109, selinexor (presenting an N-azolylacrylate scaffold, as most of the other SINEs), eltanexor and the other SINEs) covalent inhibitors and non-covalent inhibitors (NC-1, KL1 (16R), KL2 (16S), zafirlukast, B28 (R_1_= CH_3_ in *meta* and *ortho*, R_2_= Br in *para*, R_3_= H), G1 (R_1_= CF_3_ in *meta* and *ortho*, R_2_= Cl in *para*, R_3_= H), G8 (R_1_= CF_3_ in *meta* and *ortho*, R_2_= Br in *para*, R_3_= H) and G9 (R_1_= CF_3_ in both *meta* positions, R_2_= Br, R_3_=H)). The OHC mechanism is yet to be determined.

Moreover, in several cases, cancer patients show mutations in XPO1 that alter the presence of Cys528. This reflects a reduction in the efficacy of Cys528-selective drugs, leading to resistance to SINEs and, in general, to covalent Cys-targeting binders. Therefore, the development of XPO1 inhibitors effective also against Cys528 mutants became an attractive perspective. Indeed, in 2021, NCI-1, the first non-covalent XPO1 inhibitor, was presented. It was designed by combining the dimethylbutyl group of CBS9106 and the (3,5-bis(trifluoromethyl)phenyl)sulfinyl group of LFS-829, a different XPO1 inhibitor. However, since NCI-1 has a Michael acceptor group, it can also covalently bind XPO1 if Cys528 is not mutated. NCI-1 showed anticancer activity towards various cancer cell lines and also against XPO1-Cys528Ser-transfected HeLa cells, which are resistant to covalent inhibitors [133]. Two aminoratjadone derivatives, KL1 and KL2, and zafirlukast (ZAF) have been discovered in this context [84, 134, 135]. KL1 and KL2 do not contain any Michael acceptor group; thus, they cannot covalently bind Cys528 in XPO1. However, unlike NCI-1, they show sensitivity to the Cys528Ser mutation, probably due to the fact that serine is a polar amino acid, while Cys528 was predicted to be involved in hydrophobic interaction with these compounds. KL1 and KL2 showed anticancer activity against HeLa and colorectal cancer cell lines such as HCT116 and RKO [135]. ZAF was identified as an XPO1 non-covalent inhibitor in a drug repurposing study involving both *in silico* and *in vitro* approaches. ZAF, as in the case of KL1 and KL2, does not contain any Michael acceptor group, and thus cannot covalently bind Cys528. However, unlike the aminoratjadone derivatives, ZAF is insensitive to the Cys528Ser mutation, as in the case of NCI-1. Moreover, ZAF showed anticancer activity against HGC27 cells, a human gastric carcinoma cell line [136]. In a recent study, Li and colleagues [91] identified various non-covalent XPO1 binders characterized by the same scaffold. Interestingly, they noted that one of those compounds, B28, bound XPO1 in a different site, a hidden pocket, with a binding mode never observed in other XPO1 inhibitors. Indeed, it was noted that B28 bound the aforementioned pockets φ3, φ2 and φ1*, while usually XPO1 non-covalent ligands bind at least three pockets between φ0, φ1, φ2, φ3, and φ4 [91, 133]. However, B28 did not show anticancer activity against HeLa cells, leading to the synthesis of B28 derivatives based on SAR, of which three, G1, G8, and G9, showed anticancer activity towards various cancer cell lines [91]. SAR analyses revealed that in ring A, *meta,* and *ortho* substitutions were optimal since they allow the interaction with φ2 (*ortho* position) and φ3 (*meta* position). It was also noted that in these positions, bulky moieties were preferable over smaller ones. Considering ring B, they found that *ortho* substitutions were not favorable and that the best substituents were Br and Cl in *para* position, allowing molecules insertion in φ1* (Fig. **3**) [91]. The development of non-covalent ligands, besides their efficacy in the case of Cys528 mutations, might also be helpful to reduce side effects, which are problematic also for reversible covalent inhibitors, such as SINEs. Nevertheless, more studies are needed to clarify this point.

## 3 KARYOPHERIN-MEDIATED TREATMENTS: FUTURE PERSPECTIVES

Most of the abovementioned evidence was supported by *in silico* studies. During the years, several structural experiments done by X-ray diffraction, nuclear magnetic resonance (NMR), and Cryo Electron Microscopy (Cryo-EM) have allowed to obtain structural information about importins, exportins, cargoes, and their respective ligands. In this context, several records were added in the Protein Data Bank repository (https://www.rcsb.org/), and this information led to the unraveling of some features about their binding modes. Furthermore, ligand-based studies were carried out, allowing to expand the knowledge about possible derivatives of covalent and non-covalent binders for importins and exportins. In this context, SAR studies were pivotal for increasing the possibility of finding reliable candidates. Promising compounds such as eltanexor are being investigated in the field of PD. Despite the high number of limitations in NDDs drug discovery, such as BBB permeability, susceptibility to P-gp, and molecular weight, several research groups are attempting to expand the knowledge about the selectivity of these molecular targets to resolve brain pathological conditions. A family of proteins for which the mechanisms of nuclear transport have been reported to be Kap-mediated and to be involved in the regulation of their activity is one of phosphodiesterases (PDEs). In particular, this has been reported for PDE1A and PDE4D5 [137, 138] and it is under investigation for PDE9 [139], which is one of the appealing targets for epilepsy [140], AD [141], Huntington disease [142], vascular dementia [143], breast tumors [144], and prostate cancers [145]. In a more detailed manner, PDE9 is reported to have several isoforms, whereas some are exclusively cytoplasmic, nuclear, or dual [146]. This different localization was attributed to the sequence, and in the case of isoform PDE9A1, an NLS region, named Pat7, was found. Interestingly, PDE9A6/13 does not contain any NLS, and an exclusive cytoplasmic localization was reported. These features could be attributed to different causes, but the possibility that PDE9A1 could be a cargo for importins or exportins cannot be ruled out. Combined, these pieces of evidence pave the way for investigating the possible Kap/PDE9 interaction in more detail and possibly identify a new class of selective ligands given the relevant biological role of PDE9 in cancer and NDDs.

## CONCLUSION

This mini-review aimed to highlight the emerging role of drugs targeting the Kap-shuttle system in the context of CNS diseases, including cancers and NDDs.

Given the relevance that the Kap-shuttle system has at the level of intracellular regulation, several studies are attempting to identify and characterize possible Kap families important for CNS biology. With this in mind, the search for new small molecules and peptides has gained momentum in recent years, leading to the initiation of important clinical trials for anti-tumor drugs. Concerning NDDs resolving drugs, more studies are needed to identify new molecules within the CNS drug chemical space, which nowadays is still not fully explored. Although the majority of discovered ligands imply a treatment for peripheral targets, some of them are being investigated for CNS applications, as in the case of brain tumors (*e.g*., GBM) and NDDs (*e.g*., PD and amyotrophic lateral sclerosis and frontotemporal dementia) [147]. Further, with the continuous advancement in artificial intelligence-based methods, the landscape of drug discovery has transformed, particularly through significant progress in 3D protein structure prediction and the CNS-chemical space expansion. Key aspects of structure-based studies, such as the accuracy of predicted models (*e.g*., AlphaFold 3) [148], as well as the speed and cost-efficiency of calculations, have significantly expanded and enriched the chemical space of CNS-targeting molecules [149]. All these elements contribute to achieving results with greater precision and reliability. In the intricate world of karyopherin-dependent systems, where each small piece of the puzzle plays a crucial role, computational studies may hold the key to developing and optimizing kap-selective drugs, paving the way for entirely new categories of therapeutics. Indeed, future research efforts should be directed to a deeper exploration of this target category with the aim of finding new CNS-selective drugs.

## Figures and Tables

**Table 1:** Overview of importins, their isoforms and some of their related cargoes known in the literature.

Kap	Other Names	Isoforms	Cargoes
Importin-β (IMPβ)	Kapβ1, KPNB1, IPO1, IMB1, IPOB, p97, NTF97	2	Many cargoes through the adaptors Impα1 and Snurportin-1, SREBP-2, NFκB, TFEB, PD-L1
Transportin 1/Karyopherin-β2 (KAPβ2)	Impβ2, KPNB2, IPO2, TNPO1, TRN1	3	FUS, hnRNP-A1, TDP-43, β-catenin, BAP1
Transportin 2/Karyopherin-β2b (KAPβ2b)	Impβ2b, KPNB2B, IPO3, TNPO2, TRN2	2	hnRNP A1, TDP-43, FUS
Transportin 3/Transportin-SR (TNPO3)	TrnSR, TrnSR2, Trn3, Imp12, IPO12	4	ASF/SF2, CPSF6, CIRBP, HIV virus
Importin 4 (IPO4)	RanBP4, Imp4, Imp4b	2	Histones H3 and H4, Vitamin D receptor
Importin 5 (IPO5)	Impβ3, Kapβ3, RanBP5, KPNB3, Imp5, IMB3	3	Ribosomal proteins, histone H3, influenza A virus RNA polymerase subunit PB1
Importin 7 (IPO7)	RanBP7, IPO7	1	Histone H1, HIV reverse transcription complex (RTC), SMAD1, Yap1, DNA plasmids
Importin 8 (IPO8)	RanBP8, Imp8	2	cap-free eIF4E, SMAD4, microRNAs
Importin 9 (IPO9)	RanBP9, Imp9	1	ARID3A, PFKP, histones H2A and H2B
Importin 11 (IPO11)	RanBP11, Imp11	2	BZW1/2, β-catenin, PTEN

*Abbreviations: SREBP-2: Sterol regulatory element-binding proteins 2; TFEB: Transcription factor EB; PD-L1: Programmed death-ligand 1; FUS: Fused in sarcoma; hnRNP-A1:Heterogeneous nuclear ribonucleoprotein A1; TDP-43: Transactive response DNA binding protein 43 kDa (TAR DNA-binding protein 43; BAP1: BRCA1 associated protein 1; ASF/SF2: Serine/arginine-rich splicing factor 1; CPSF6: Cleavage And Polyadenylation Specific Factor 6; CIRBP: Cold-inducible RNA-binding protein; HIV: human immunodeficiency virus; SMAD1: Suppressor of mother against decapentaplegic; YAP1: yes-associated protein 1; elF4E: Eukaryotic translation initiation factor 4E; ARID3A: AT-rich interactive domain-containing protein 3A; PFKP: Phosphofructokinase, platelet; BZW1/2: Basic Leucine Zipper And W2 Domains 1; PTEN: Phosphatase and Tensin Homolog.

**Table 2:** Overview of exportins, their isoforms and some of their related cargoes known in the literature.

**Kap**	**Other Names**	**Isoforms**	**Cargoes**
Chromatin Region Maintenance 1/Exportin 1 (CRM1/XPO1)	Exp1, emb	1	p53, FOXO, Survivin, TFEB, cGAS
Cellular Apoptosis Susceptibility/Exportin 2 (CAS/XPO2)	Exp2, CSE1L, Impα re-exporter	4	Impα1-7
Exportin-t (XPOT)	Exp-T, XPO3	1	aminoacylated tRNAs
Exportin 5 (XPO5)	Exp5, RanBP21	1	Pre-miRNA, tRNA, hairpin RNA, RNA,and co-exported proteins
Exportin 6 (XPO6)	Exp6, RanBP20	2	Nuclear actin

*Abbreviations: p53: tumoral protein 53; FOXO: Forkhead box O3; cGAS: Cyclic GMP-AMP synthase.

**Table 3:** Overview of biportins, their isoforms and some of their related cargoes known in the literature.

**Kap**	**Other Names**	**Isoforms**	**Cargoes**
Importin 13 (IPO13)	Kap13, RanBP13, IPO13	1	**Import cargo**: Mago-Y14, Ubc9 **Export cargo**: eIF1A
Exportin 4 (XPO4)	Exp4	1	**Import cargo**: Sox2, SRY **Export cargo**: eIF5A1, eIF5A2, Smad3
Exportin 7 (XPO7)	Exp7, RanBP16	1	RhoGAP1, 14-3-3σ, p65

*Abbreviations: Mago-Y14: Mago nashi; Ubc9: ubiquitin conjugating enzyme; Sox2: SRY-Box Transcription Factor 2; SRY: Sex -determining region Y protein; RhoGAP1: Rho GTPase-activating protein 1, p65: nuclear factor NF-kappa-B p65 subunit.

**Table 4:** Overview of unknown karyopherins and their isoforms known in the literature.

**Kap**	**Other Names**	**Isoforms**	**Cargoes**
RANBP6	NA	1	Putative cargo: STAT3
RANBP17	NA	2	-

*Abbreviations: STAT3: Signal Transducer and Activator of Transcription 3.

## LIST OF ABBREVIATIONS

AP-1: Activator Protein 1
AD: Alzheimer’s Disease
AR: Androgen Receptor
ABC: ATP-binding Cassette
BBB: Blood-brain Barrier
BCRP: Breast Cancer Resistance Protein
CREBBP: cAMP Response Element-binding Protein
Felezonexor: CBS9106
c-MYC: Cellular Myelocytomatosis Oncogene
CNS: Central Nervous System
CRM1/XPO1: Chromatin Region Maintenance 1
Cryo-EM: Cryo Electron Microscopy
E2F1: E2 Transcription Factor 1
XPO: Exportins
FDA: Food and Drug Administration
FOXM1: Forkhead Box Protein M1
FOXO1: Forkhead Box Protein O1
DD1: Genkwadaphnin
GBM: Glioblastoma
HIV-1: Human Immunodeficiency Virus Type 1
HEAT: Huntingtin, Elongation Factor 3, Protein Phosphatase 2A, and TOR1
HBA: Hydrogen Bond Acceptors
HBD: Hydrogen Bond Donors
HIF1α: Hypoxia-inducible Factor 1 Subunit α
IPZ: Importazole
IBB: Importin β Binding
IMP/IPO: Importins
IDPs: Intrinsically Disordered Proteins
Kap: Karyopherin
Selinexor: KPT-330
Verdinexor: KPT-335
Eltanexor: KPT-8602
LMB: Leptomycin B
MoA: Mechanism of Action
MRP: Multidrug Resistance Protein
MDM2: Murine Double Minute 2
MD2: Myeloid Differentiation Protein 2
MTPT: 1-methyl-4-phenyl-1,2,3,6-tetrahydropyridine
NDDs: Neurodegenerative Diseases
IκB-α: NF-κB Inhibitor
NS5: Non-structural Protein 5
NESs: Nuclear Export Sequences
NF-κB: Nuclear Factor Kappa-light-chain-enhancer of Activated B Cells
NFAT: Nuclear Factor of Activated T-cells
NLSs: Nuclear Localization Sequences
NMR: Nuclear Magnetic Resonance
NPCs: Nuclear Pore Complex
OHC: Octahydrocurcumin
PD: Parkinson’s Disease
PARK7: Parkinsonism-associated Deglycase
P-gp: P-glycoprotein
PDEs: Phosphodiesterases
PSA: Polar Surface Area
PCNA: Proliferating Cell Nuclear Antigen
PIASy: Protein Inhibitor of Activated STAT Y
p107: Retinoblastoma-like Protein 1
Rio2-kinase: Right Open Reading Frame 2 Protein Kinase
SINEs: Selective Inhibitors of Nuclear Export
STAT1: Signal Transducer and Activator of Transcription 1
STAT3: Signal Transducer and Activator of Transcription 3
SAR: Structure-activity Relationship
ZAF: Zafirlukast
